# Experimental Investigation of Integrated Circular Triple-Wire Pulse GMAW of Q960E High-Strength Steel for Construction Machinery

**DOI:** 10.3390/ma14020375

**Published:** 2021-01-14

**Authors:** Ke Yang, Fei Wang, Dingshan Duan, Tianli Zhang, Chuanguang Luo, Yann Cressault, Zhishui Yu, Lijun Yang, Huan Li

**Affiliations:** 1 Tianjin Key Laboratory of Advanced Joining Technology, Tianjin University, Tianjin 300072, China; swsyangke@tju.edu.cn (K.Y.); yljabc@tju.edu.cn (L.Y.); 2 School of Materials Engineering, Shanghai University of Engineering Science, Shanghai 201620, China; wangfei@sues.edu.cn (F.W.); zhangtianli925@163.com (T.Z.); yu_zhishui@163.com (Z.Y.); 3 Shanghai Collaborative Innovation Center of Laser Advanced Manufacturing Technology, Shanghai 201620, China; 4 ROFS Microsystem (Tianjin) Co., Ltd., Tianjin 300462, China; duan_dingshan@163.com; 5 Sichuan Institution of Aerospace Systems Engineering, Chengdu 610100, China; chg_luo@163.com; 6 LAPLACE (Laboratoire Plasma et Conversion d’Energie), UPS, INPT, Université de Toulouse, 118 route de Narbonne, 31062 Toulouse, France; cressault@laplace.univ-tlse.fr

**Keywords:** Q960E, multi-wire welding, arc interference, high strength steel, phase control

## Abstract

Multi-wire welding has received much attention in the machinery industry due to its high efficiency. The aim of this study was to investigate a novel pulse gas metal arc welding (GMAW) that has circular triple-wire electrodes. The effect of the pulse phage angle on arc stability was particularly studied. Research showed that for typical phase angles the arc stability from low to high is 180°, 0°, and 120°, and the arcs are very stable at 120°. The triple-wire welding was used to weld a 9 mm thick Q960E steel, which is typically used for the arm of construction machinery. When the welding heat input was controlled at 1.26–1.56 kJ/mm, the weld zone was dominated by acicular ferrite, and the coarse-grained zone of the heat-affected zone was a mixed structure of lath martensite and lath bainite. The tensile strength of the welded joint reached 85% of the base metal and the impact toughness was above 62 J, which can meet the requirements of construction machinery. This indicates that the triple-wire welding has great potential to achieve efficient and high-quality welding for the construction machinery.

## 1. Introduction

Welding is an important material processing technology in modern industrial production, and the improvement of welding efficiency plays an important role in the improvement of total productivity [1]. With the advantages of high quality and low cost, gas metal arc welding (GMAW) is now one of the most dominant methods of welding. In the process, GMAW utilizes an electric arc to melt two pieces of metals, and the metals are joined together after cooling. How to improve the efficiency of the GMAW process has always been a key issue in welding research [2,3]. High welding efficiency mainly reflects a high deposition rate or high welding speed [4].

There are several alternatives for raising the efficiency of GMAW process, such as using direct current electrode negative (DCEN) operation, extended electrical stick out, high operating current, or a combination of these effects [5,6,7,8]. Operating GMAW with DCEN can increase the burn off rate of the wire by up to 50%, but the electric arc behaves erratically [9]. The extension of electrical stick out will enhance the deposition rate due to the increased resistive heating of the wire, but this may affect the protection effect of the shielding gas [10]. The increase of welding current can enhance the melting of the workpiece and wire, but too large a current will cause excessive arc force, unsteady droplet transfer, and poor surface formation [11]. In the 1980s, Canadian researchers proposed a high-efficiency GMAW technology—Transferred Ionized Molten Energy (T.I.M.E.) welding, which adopts high current, large stick out, and a 65%Ar-26.5% He-8%CO_2_-0.5%O_2_ quaternary shielding gas. T.I.M.E. welding can achieve superior welding with a high deposition rate of above 10 kg/h [10], but it relies on costly shielding gas, surface finished wire, and precise welding power and wire feeder.

The recent development in high-productivity GMAW has been the use of multi-wire welding systems. As its typical representative, TANDAM welding involves two wires sharing a common gas shield in which the two wires are operated from independent power sources, and it can achieve a deposition rate of 12 kg/h without the flaw of undercut because of a more homogeneous weld pool temperature [12,13]. To further increase the GMAW efficiency, researchers have developed various triple-wire GMAW processes. Arita et al. [14] presented a triple-wire horizontal fillet GMAW process where a filler wire was additionally placed between the leading and trailing wires and was electrified with DCEN, but no arc occurs. It has been proven that the centrally filler wire has a stabilizing effect on the arcs, and the deposition rate could be increased [15]. Hua et al. [16] developed a high-speed triple-wire GMAW process where three wires were arranged longitudinally and operated with independent gas shield, power supplies, and wire feeders. The welding parameters, wire spacing and torch angle were flexibly adjusted to adopt different welding conditions, and the welding speed can achieve 1.8 m/min [17,18]. The problems of arc interruption and hump were also studied by Hua’s research group [19,20,21,22]. Fang et al. [23] proposed a triple-wire indirect GMAW process, which consists of one main wire, two side wires and two power sources. In the system, the workpiece never needs to be plugged in, while the main wire and the two wires were connected with the negative pole and the positive pole of the two power sources, respectively. This method has been successfully used in narrow gas welding [24,25].

Based on an understanding of the multi-wire welding systems and long-term practical experience, we have developed a novel welding method—integrated circular triple-wire GMAW. In the welding process, three wires are circularly integrated in a big torch and powered by three pulse power sources with cooperative control function respectively. This triple-wire configuration makes the arc heating and welding wire filling more concentrated. By using it, we have welded Q235 steel with a deposition rate of 15 kg/h [26,27], which is higher than that by using tandem GMAW.

Although the triple-wire welding can significantly improve welding efficiency, the interference between the arcs is serious, which may lead to the instability of the welding process. The arc interference is related to the control of pulse currents, and the control of pulse currents depends on the choice of phase angle (see its definition in Equation (1)). Therefore, the present study particularly investigates the influence of pulse phase angle on arc stability and aims to find the best phase angle. This study also deals with expanding the application of the triple-wire welding to high-strength steel because the construction machinery filed prefers to use higher-strength steel (e.g., Q960E) to replace Q235/Q345 as structural material for energy saving. For example, the use of Q960E steel in excavators can reduce weight by more than 60% and therefore reduce fuel consumption by 40% [28,29]. At present, the welding of Q960E steel mainly depends on single wire GMAW [30,31], which seriously restricts product productivity. Therefore, the triple-wire welding was expected to achieve an efficient and high-quality welding for construction machinery. Since heat input is a key influence parameter for joint microstructure and performance, the influence of heat input on weld joints was investigated for a 9 mm thick Q960E plate, which is typically used for the arm of excavators or hydraulic supports, and a satisfactory joint was obtained when using a value of 1.26–1.56 kJ/mm.

## 2. Experiments

### 2.1. Experimental Setup and Materials

**Figure 1:**
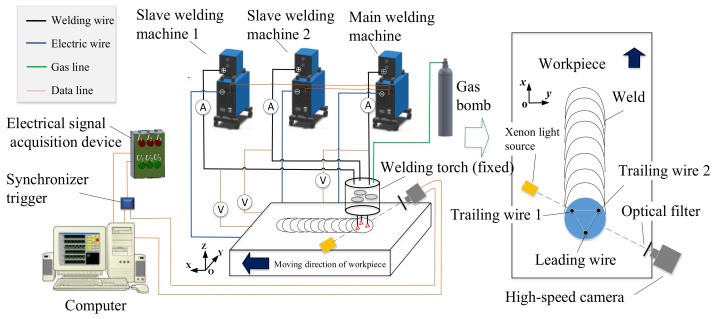
Schematic diagram of experimental setup.

Figure 1 shows a schematic diagram of the experimental setup. The experimental setup consisted of four parts: (1) a welding system, including three Aotai Pulse MIG-500 welding machines (Aotai Electric Co., LTD., Jinan, China), a special three-wire welding torch, and a welding bench; (2) a high-speed camera system, including a MOTION PRO high-speed camera (DEL Imaging Systems, LLC., Woodsville, FL, America) and a xenon light source (Microenerg Beijing Technology Co., Ltd., Beijing, China) [32]; (3) an electrical signal acquisition device, including a NI USB6251 data acquisition card, three LEM Hall current sensor, and three LEM Hall voltage sensors; and (4) a data processing system, including a synchronous trigger device and a computer with data processing software.

The key devices of the welding system were the special welding torch and the three welding machines with communication function. As shown in Figure 1, three welding wires are insulated from each other and circularly arranged with an equilateral triangle configuration in a big welding torch. The side length of the equilateral triangle was 10 mm. In order to facilitate the shooting of the welding arc, the welding torch was fixed while the workpiece was moving. Hence, the welding direction was opposite to the moving direction of the workpiece. The welding wire close to the front end of the molten pool was called a guide wire, and the two wires located at the rear end of the molten pool were called trailing wire 1 and trailing wire 2.

**Figure 2:**
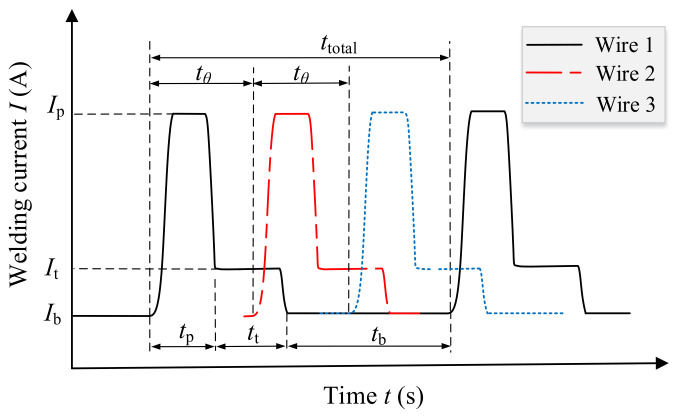
Schematic diagram of welding current waveform for the triple-wire welding.

The three wires were powered by the three welding machines respectively. All the arcs on the wires were operated in pulse mode, which is conducive to realize one pulse per one drop of molten droplets. The cooperative control of the three pulse currents depended on the main welding machine, which sends delay signals to the remaining two welding machines. The other two machines are referred to as slave welding machines. The time relationship of the three pulse currents is represented by the phase angle *θ*, which is defined as follows:

*θ* = 360°·*t*_θ_/*t*_total_ (1)

where *t*_θ_ is the delay time of the current pulse between the wire 1 and wire 2. Wire 2 and wire 3 adopt the same delay time. *t*_total_ is the total time of a current pulse cycle (see Figure 2).

There are three typical phase angles: 0°, 120°, and 180°. When the phase angle is 0°, the pulse peak phase appears on the three welding wires simultaneously; when the phase angle is 120°, the pulse peak phase appears alternately on the three welding wires, whose state is the same as that shown in Figure 2; when the phase angle is 180°, the pulse phase first appears on the leading wire (wire 2), and after 0.5·*t*_total_ it appears on the two trailing wires (wire 1 and 3) simultaneously.

The pulse current waveform was improved on the basis of the traditional rectangular wave by adding a platform of transition current *I*s, as shown in Figure 2. The addition of the current platform has two advantages: First, it reduces the arc interruption caused by the sudden change of current [33]; second, it is beneficial for the droplet transfer because there is still a large electromagnetic contraction force, before the current turns to the base value [34]. For any single wire, all the pulse waveform parameters were fixed except for the base time (see Table 1), and thus the average welding current as well as the pulse frequency was determined only by the base time.

The Q960E high-strength steels produced by Wuyang Iron and Steel Co., Ltd. (Pingdingshan, China) were chosen as the test piece, whose chemical compositions are shown in Table 2. After quenching and tempering, the microstructure of these steels is sorbite and the yield strength is 1015 MPa. Considering that Q960E steel has some cold crack sensitivity, we chose THQ80-1 wire with relatively low strength as the filler wire, according to the principle of “low-strength matching” [3]. The yield strength of the THQ80-1 wire was about 730 MPa, and its chemical compositions are also given in Table 2. The diameter of the three welding wires is 1.2 mm. The 82% Ar-18% CO_2_ gas mixture, which is widely used for steel welding, was chosen as the shielding gas.

The high-speed camera system was used to take photos for the welding arc and the molten droplets. The sampling frequency of the high-speed camera (MOTION PRO) was set to 2000 frames/s. The shadow method [35] was used for shooting; that is, a high-brightness xenon light source was used as a backlight to form the shadow of droplets. It was also necessary to place a neutral filter in front of the camera to avoid overexposure. The high-speed camera pictures were transmitted to the computer via a data cable.

The electric signal acquisition system was used to record the welding current and arc voltage. The welding current and arc voltage information for each wire were obtained by the current sensor and voltage sensor respectively, and then they were transmitted to a NI USB6251 data acquisition card [32]. The sampling frequency of the data card was 100 kHz/s, and the measure error was less than 5%, which is acceptable for this study. The data collected by the data acquisition card is transmitted to a computer, and displayed by a LabVIEW program. In order to obtain the high-speed camera pictures and electrical signals simultaneously, a synchronous triggering device was used to trigger the two devices by sending out a 5 V high-level voltage to them at the same time.

### 2.2. Experimental Procedure

First, the influence of phase angle on arc stability was studied. In order to better observe the arc, we adopted the form of bead-on-plate welding. Three typical phase angles of 0°, 120°, and 180° were chosen. The same preset current of 140 A and preset voltage of 24.0 V were used for the three welding wires. This voltage current matching facilitates the realization of free transition of metal droplets. Detailed welding parameters are given in Table 3.

Next, the influence of welding heat input on joint quality was studied. The butt welding was performed to weld a Q960E steel plate with a 9 mm thickness, which is typically used for the arm of excavators or hydraulic supports. A 60° V-shaped groove was adopted to facilitate full penetration of the weldment. In order to avoid the formation of a hardened microstructure and reduce welding stress and deformation, we preheat the weldment to 120 °C before welding according to the suggestion of [31]. Single-wire metal active gas (MAG) welding was first used for backing with a preset current of 185 A. Then, the triple-wire welding was used to achieve filling and cosmetic welding. For the phase angle, we set it to 120°, because the first study showed that at this angle the arc interference is the weakest and the welding process is the most stable. From low to high values, four heat input conditions were used to finish the triple-wire butt welding, as shown in Table 4. The heat input *E* infers to the welding energy to the weld per unit length. It can be expressed as:

*E* = n*I_set_*U_set_/*υ* (2)

where *n* is the number of welding wires (*n* = 3 in this case), *I*_set_ is the welding current, *U* is the welding voltage, and *v* is the welding speed. For example, for the test number 1, the heat input is 3 × 120 × 22/8/10^3^ = 0.99 kJ/mm.

After welding, the microstructures of the welded joints were examined by an OLYMPUS DP70 optical microscopy (Olympus Corporation, Tokyo, Japan). The mechanical properties of the welding joints including tensile strength and impact toughness were also examined. The tensile test was carried out on a DDL 300 electronic universal testing machine (CIMACH, Changchun, Jilin, China), according to the standard of ISO 6892: 1998 (Metallic Materials-Tensile testing at ambient temperature) [36]; the Charpy-V notch impact test was conducted on a ZBC2752-ED impact testing machine (MTS, Beijing, China) at −40 °C, according to the standard of ISO 9016: 2001 (Impact test methods on welded joints) [37]. The notch positions were chosen in the center of the weld and in the heat-affected zone, respectively.

## 3. Results

### 3.1. Influence of Pulse Phase Angle on Arc Stability

**Figure 3:**
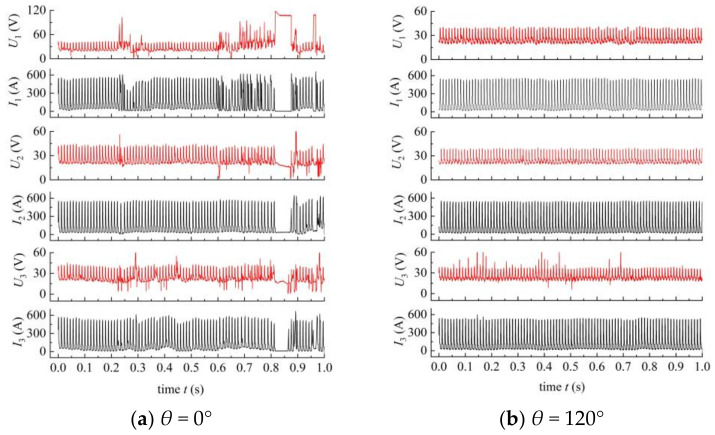

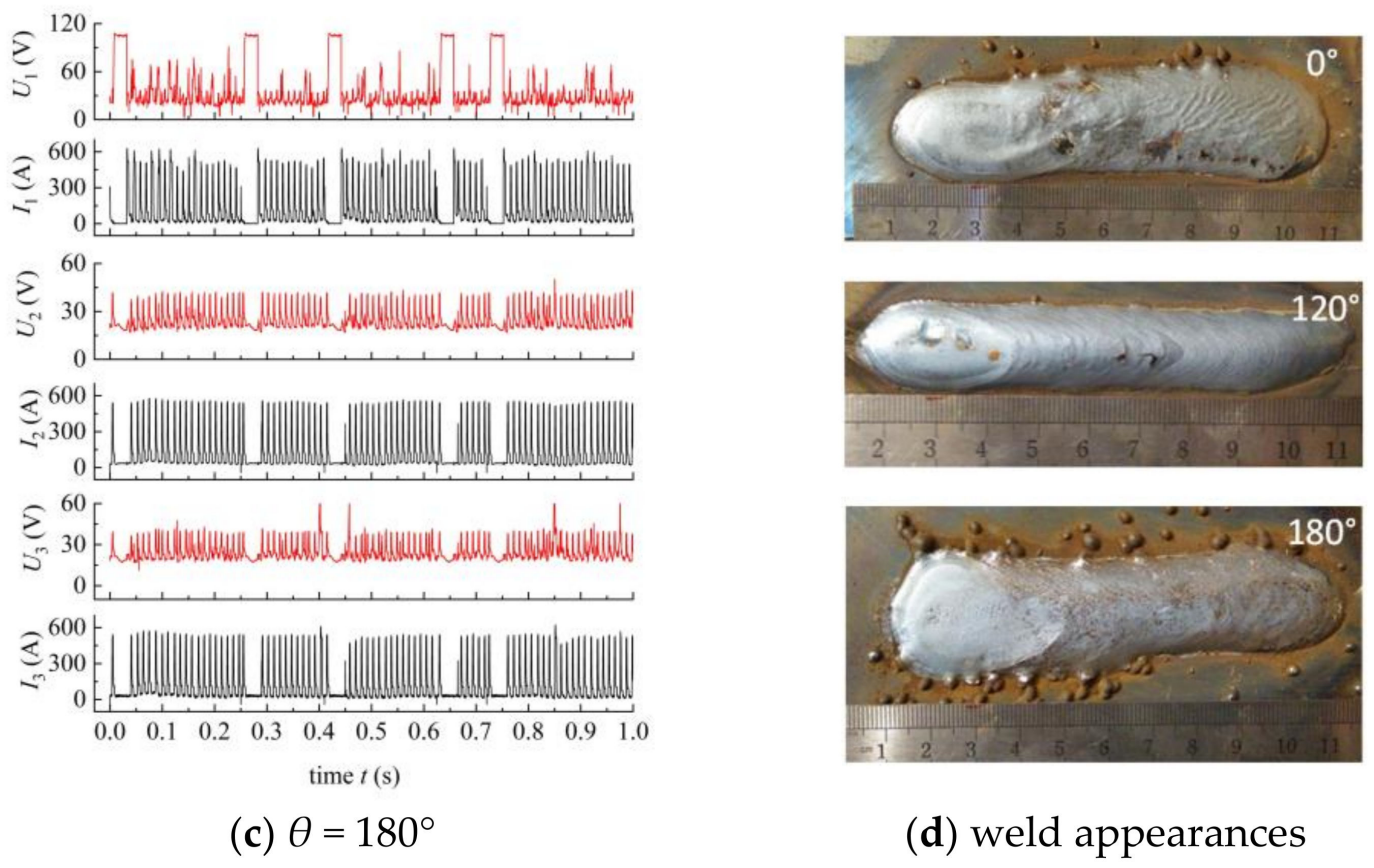
Arc voltage and welding current waveforms (**a**–**c**) and weld appearances (**d**) for the phase angles of 0°, 120°, and 180°.

Welding electrical signals, especially the arc voltage signal, can intuitively reflect whether the welding arc is stable or not [38]. When the arc is unstable, such as large arc offset or arc interruption, the arc voltage will show abnormality, so we can judge the stability of the arc based on the electrical signals. Figure 3 shows the changes in arc voltage and welding current (a, b, c) and the weld appearances (d). When the phase angle was 120°, the arc voltage signal was very regular, and there was no abnormal voltage (Figure 3b), indicating that the welding process was very stable. When the phase angle was 180°, the three welding wires, especially the guide wire, had many abnormal voltages (Figure 3c), indicating that the welding process was unstable. When the phase angle was 0°, the probability of arc interruption was less than that at 180° (Figure 3a), indicating that its arc stability was stronger than that at 180° but worse than 120°. The stability of the arc was also reflected in the weld appearance. As shown in Figure 3d, the weld forming quality from good to bad for the three phase angles was as follows: 120°, 0°, 180°; it is consistent with the result reflected by the electrical signals.

**Figure 4:**
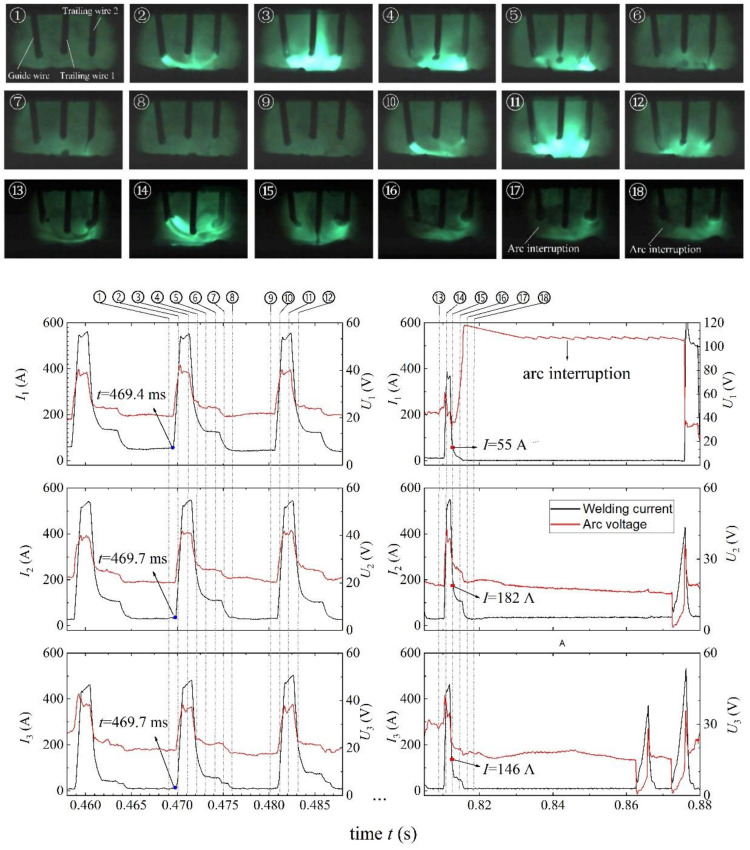
Typical arc images for the 0° phase angle.

**Figure 5:**
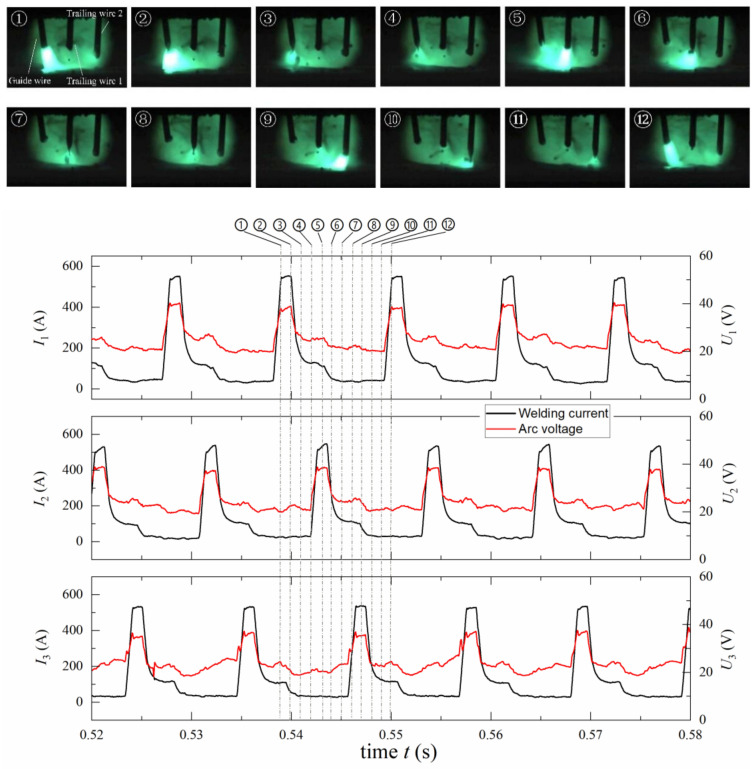
Typical arc images for the 120° phase angle.

**Figure 6:**
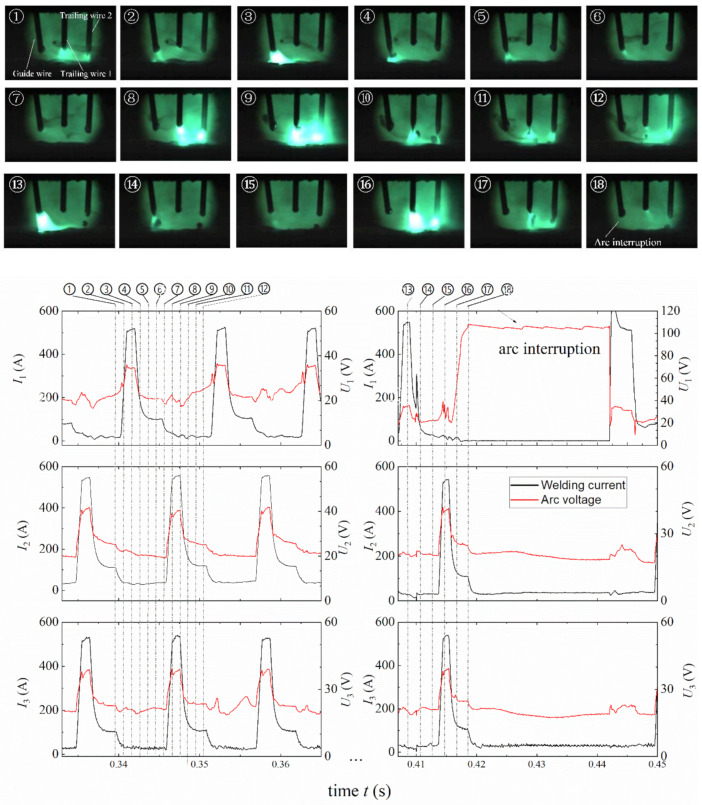
Typical arc images for the 180° phase angle.

In order to better illustrate the arc behavior of the triple-wire welding, typical welding arcs in stable and unstable states are presented for the phase angles of 0°, 120° and 180° in Figure 4, Figure 5 and Figure 6.

Figure 4 shows typical arc images for the 0° phase angle in stable and unstable arc states. During 0.458–0.488 s, the welding arc was relatively stable. Three bright pulse arcs were generated on the three welding wires synchronously. Due to the electromagnetic effect, three arcs were close to each other. The metal droplets were transferred with one-pulse-one-drop mode and dropped close to the center of the three arcs. In the pulse base stage, the three arcs became dim, and they were even invisible in the background of xenon lamp light. It should be noted that there was a tiny time difference of 0.3 ms in current pulse between the guide wire arc and the two trailing wire arcs due to communication delay from the main welding machine to the two slave machines. For example, a current pulse began to appear on the guide wire arc at *t* = 469.4 ms, and after 0.3 ms it appeared on the two trailing wire arcs at *t* = 469.7 ms. During 0.807–0.880 s, the welding arcs, especially the guide wire arc, became unstable. At *t* = 0.817 s, the welding current became zero ampere, and the arc voltage exceeded 100 V for the guide wire, which means its arc has been broken. Just before that, in the current fall stage, because of the pulse delay, the guide wire current was significantly smaller than the trailing wire currents, e.g., *I*_1_ = 55 A, *I*_2_ = 182 A and *I*_3_ = 146 A at *t* = 0.813 s, which directly leads to the arc interruption. A detailed discussion will be given in Section 4.

Figure 5 shows the arc images for the 120° phase angle. A luminous arc first appeared on the guide wire at *t* = 0.539 s, and the offset of the arc was not very obvious. The wire melted under the action of the arc heat to form a droplet. When entering the transition current stage, the brightness of the arc gradually became dim, and the droplet fall off with one-pulse-one-drop mode. At *t* = 0.543 s and 0.547s, the luminous arcs appeared on the trailing wire 1 and wire 2, respectively, and then it appeared on the guide wire again. Throughout the welding process, luminous pulse arcs appeared on the three welding wires alternately.

Figure 6 shows typical arc images for the 180° phase angle in stable and unstable arc states. During 0.333–0.365 s, the arc burned steadily. At *t* = 0.342 s, a luminous pulse arc appeared on the guide wire, while at the same time the two trailing wire arcs were very dim, even invisible. After 1/2 delay time, the two trailing wire arcs became bright simultaneously, and the guide wire arc became dim at *t* = 0.347 s; during 0.410–0.450 s, the arcs, especially the guide wire arc, became unstable. Since the low-current guide wire arc was strongly electromagnetically attracted by the two high-current trailing wire arcs, the arc voltage on the guide wire first fluctuated sharply, and then the arc voltage increased to about 100 V, and the current decreased to 0 A at *t* = 0.418 s, indicating that the guide wire arc has been broken.

### 3.2. Influence of Heat Input on the Microstructure and Mechanical Properties of the Welded Joints

**Figure 7:**
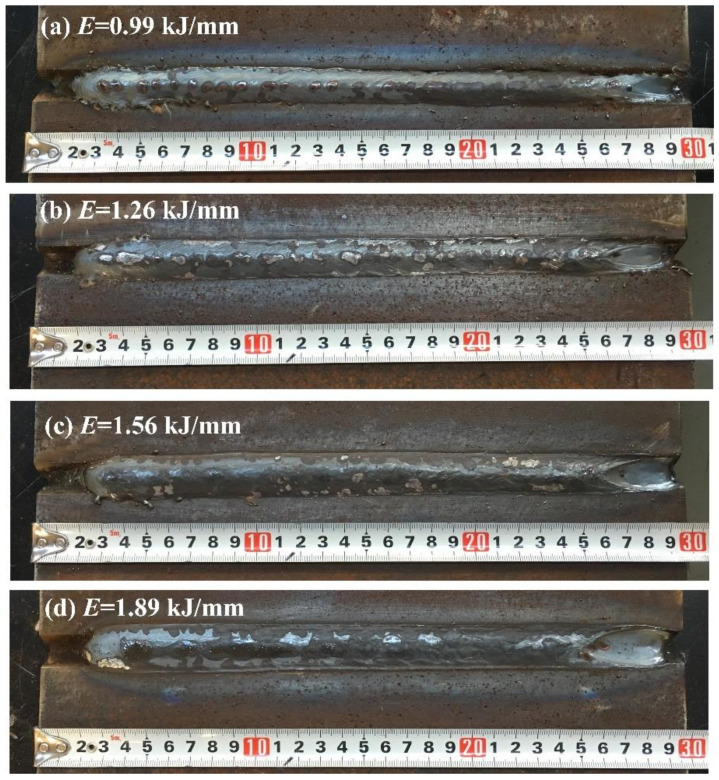
Weld appearances under different heat input values.

**Figure 8:**
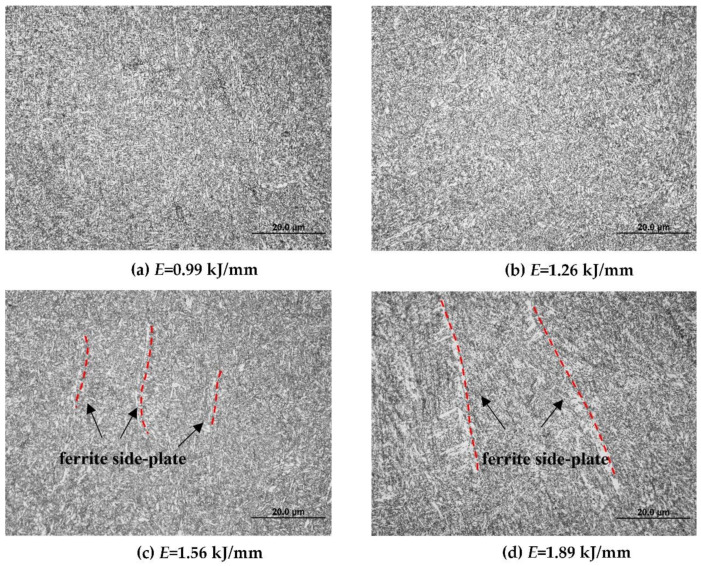
Microstructures of the weld seam under different heat input values.

Since the welding arc was stable at the 120° pulse phase angle, the Q960E plates with a thickness of 9 mm and a groove angle of 60° were welded in a butt joint by the triple-wire welding at 120°. The weld appearances under different heat input values are presented in Figure 7. Their corresponding microstructures in the weld seam are shown in Figure 8. When the heat input values were 0.99 and 1.26 kJ/mm, the weld microstructures were dominated by fine acicular ferrite, as shown in Figure 8a,b, whose grain boundaries can block the propagation of cracks and improve the toughness of the weld. When the heat input value was 1.56 kJ/mm, a small amount of ferrite side plate appeared in the weld structure, and the grain size of acicular ferrite increased, as shown in Figure 8c. When the heat input was 1.89 kJ/mm, more ferrite side plates appeared in the weld microstructure, and the size of the acicular ferrite further increased. This ferrite side plate grew into the grain in a lath shape along the austenite grain boundary, and it looks like a pick tooth in shape from the morphology, as shown in Figure 8d. Usually the ferrite side plate is formed at a temperature range of 550–700 °C, and the acicular ferrite is formed at around 500 °C. With the increase of heat input, the high temperature residence time of the welded joint increased and the cooling rate became slower, which is conducive to the grain growth and the high temperature transformation of the microstructure. As a consequence, the content of the ferrite side plate increases.

**Figure 9:**
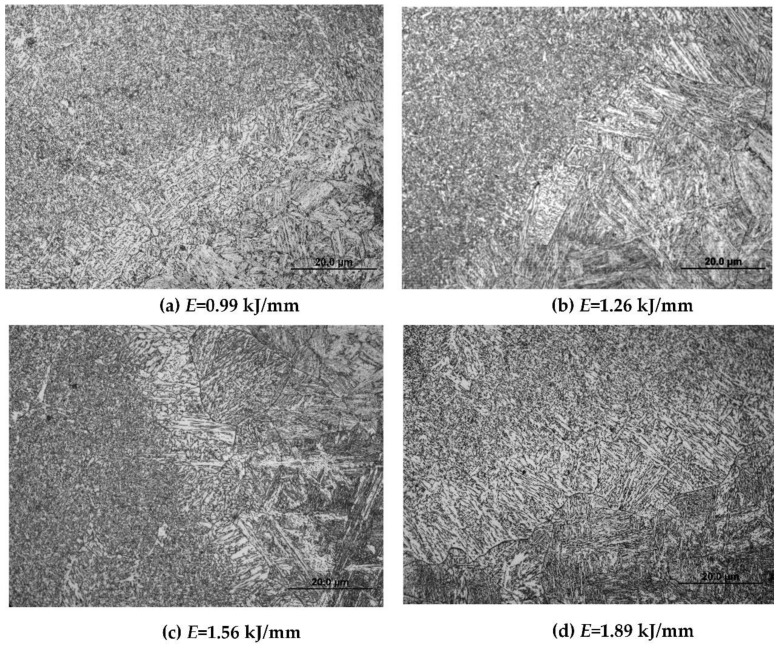
Microstructures of heat-affected zone under different heat input values.

Figure 9 shows the microstructures of the heat-affected zone under different heat input values. When the heat input was 0.99 kJ/mm, the microstructure of the coarse-grained zone was dominated by lath martensite and the grains were relatively small, as shown in Figure 9a. When the heat input was 1.26 kJ/mm, the coarse-grained zone had some lath bainite besides lath martensite, as shown in Figure 9b. When the heat input was 1.56 kJ/mm, the coarse-grained zone was a mixed microstructure of lath martensite and lath bainite, and the grain size increased significantly, as shown in Figure 9c. When the heat input increased to 1.89 kJ/mm, the heat-affected zone was mainly composed of lath bainite, as shown in Figure 9d. Therefore, with the increase of welding heat input, the grain size of welded joints increased significantly, and the microstructure of the heat-affected zone changed from lath martensite to lath martensite + lath bainite and then to lath bainite.

**Figure 10:**
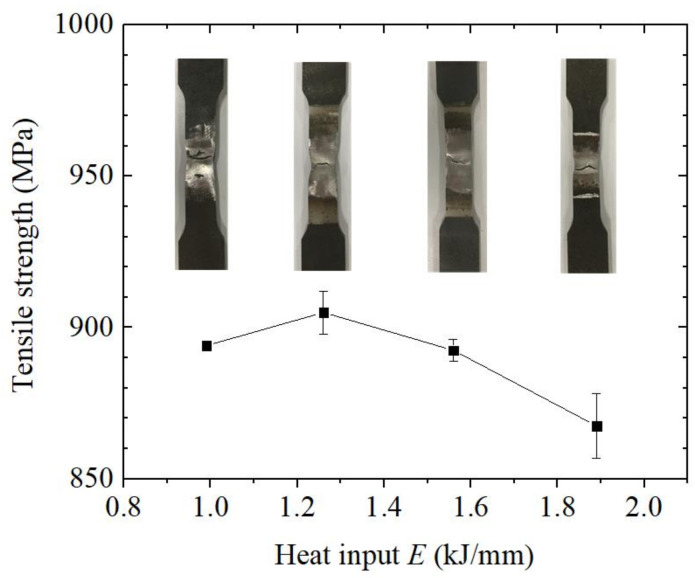
Tensile strength of the welded joints under different heat input values.

Figure 10 shows the results of the tensile test for the welded joints under different heat input values. In all the cases, the welded joints were broken at the welded seam. This is because the welded joint has been designed with the “low-strength matching” principle. For the tensile strength, it had not changed much when the heat input was between 0.99 and 1.56 kJ/mm, and it reached a maximum of 905 MPa at 1.26 kJ/mm, which is equivalent to 87% of the tensile strength of the base material. When the heat input was 1.89 kJ/mm, the tensile strength was significantly reduced. The previous metallographic experiment has shown that with the increase of heat input, the content of ferrite side plate in the weld seam increased, the acicular ferrite needles became thicker, and the number of grain boundaries relatively decreased. These elements result in the reduction of crack propagation resistance, and thus the strength of the welded joint was reduced.

**Figure 11:**
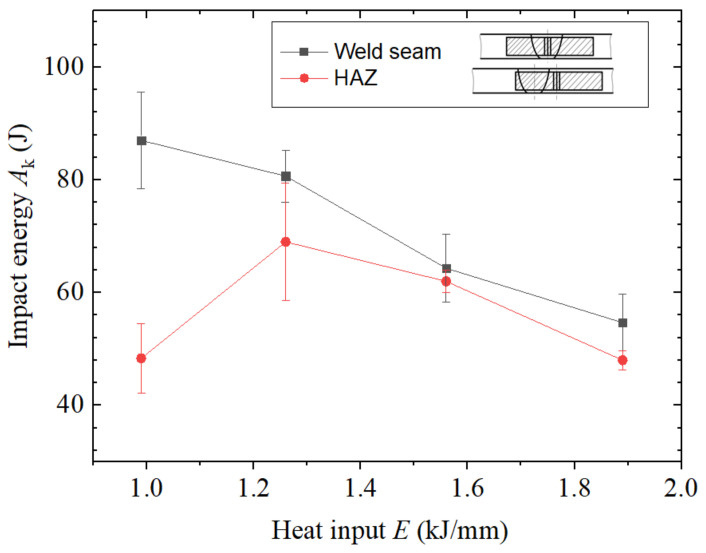
Impact toughness of the welded joints under different heat input values.

Figure 11 shows the impact toughness of welded joints under different heat input values. Under the same heat input condition, the impact toughness of the weld zone was higher than that of the heat-affected zone. With the increase of heat input, the impact toughness of the weld seam showed a downward trend, and the impact toughness of the heat-affected zone first increased and then decreased. When the heat input was 0.99–1.26 kJ/mm, the impact toughness of the weld zone was good. When the heat input was 1.26–1.56 kJ/mm, the impact toughness of the heat-affected zone was good. When the welding heat input is increased to 1.89 kJ/mm, the impact toughness of the weld zone and heat-affected zone was significantly reduced.

As a consequence, at the 120° phase angle, when the welding heat input was controlled at 1.26–1.56 kJ/mm, the structure of the weld zone was dominated by acicular ferrite, and the coarse-grained zone of the heat-affected zone was a mixed structure of lath martensite and lath bainite. The tensile strength of the welded joint reached 85% of the base metal, and the impact toughness was above 62 J, which can meet the requirements of construction machinery for joint performance.

## 4. Discussion

Section 3 has shown that the pulse phase angle had an important influence on the arc stability of the triple-wire pulse welding. When the phase angle was 120°, the welding arc was very stable, and there was no arc interruption. When the phase angle was 180° or 0°, especially 180°, the inter-arc interference phenomenon was obvious, the guide wire arc was often broken. In order to better illustrate this point, we specifically discuss it here.

When an arc is close to the other arcs, the arc will shift due to the electromagnetic forces caused by the adjacent arcs. If the arc offset is too large, the arc may be broken. The arc interruption is mainly determined by two contradictory effects: (1) The first is the stiffness of the arc itself. Arc stiffness refers to the degree to which the arc is straight along the electrode axis. The greater the arc stiffness, the less likely it is to be offset by external force. (2) The second effect is the electromagnetic action caused by adjacent arcs. The stronger the electromagnetic force, the greater the arc offset, and the easier the arc is to break.

**Figure 12:**
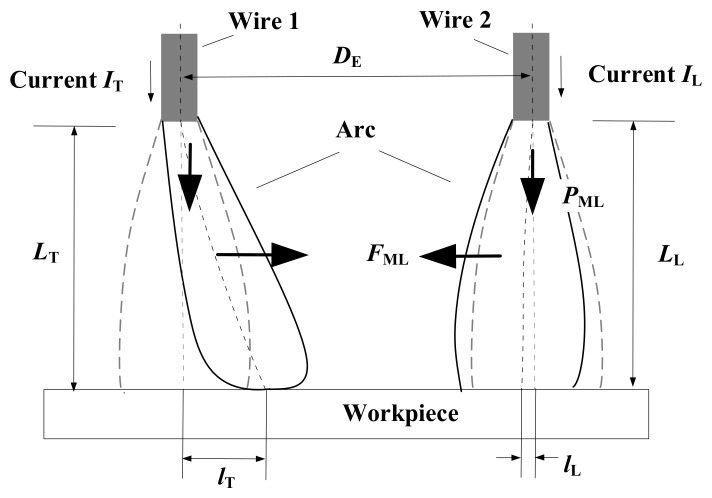
Schematic diagram of a double-wire arc model.

In order to explain the arc behavior for the triple-wire welding, the double-wire arc is first analyzed. The schematic diagram of a double-wire arc model is shown in Figure 12. Take arc 2 (the right arc) as the research object. The arc stiffness is mainly determined by the pressure difference between the electrode end and the workpiece. The greater the pressure difference, the more intense the arc flow and the greater the stiffness. The pressure difference *P*_ML_ for the right arc can be approximately expressed as [39]:

(3) $$P_{\mathrm{ML}}=\frac{\mu_{0}}{4\pi }\frac{I_{L}^{2}}{{\pi r}_{L}^{2}}$$

where $\mu_{0}$ is the vacuum permittivity, $r_{L}$ is the arc column radius, and $I_{L}$ is the welding current. According to the above formula, the pressure difference *P*_ML_ and the current square $I_{L}^{2}$ are in a certain ratio *k*, where $k=\mu_{0}/4\pi^{2}r_{L}^{2}$. It can see from the formula that the greater the current, the greater the pressure difference and the greater the arc stiffness.

For arc 2, the electromagnetic force caused by the adjacent arc 1 is *F*_ML_ = *j*_L_ × *B*_T_, where *j*_L_ is the current density, and *B*_T_ is the magnetic induction intensity generated by arc 1. The current density *J*_L_ is equal to $I_{L}/{\pi r}_{L}^{2}$, and the magnetic induction intensity *B*_T_ is equal to $\mu_{0}I_{T}/2\pi D_{E}$. After deduction, the electromagnetic force *F*_ML_ can be expressed as [39]:

(4) $$F_{\mathrm{ML}}=\frac{\mu_{0}I_{L}I_{T}}{{2\pi^{2}D}_{E}r_{L}^{2}}$$

where $I_{T}$ and $I_{L}$ are the welding currents of arc 1 and arc 2 respectively, and $D_{E}$ is the distance between the two arcs. Suppose the arc column size and the arc distance remain unchanged, and the electromagnetic force *F*_ML_ and the current product $I_{L}I_{T}$ is in a fixed ratio *m*, with $m=\mu_{0}/{2\pi^{2}D}_{E}r_{L}^{2}$. The increase of the current of the arc itself and the adjacent arc current will increase the electromagnetic force *F*_ML_.

When the current flows through arc 1 and arc 2 in the same direction, the two arcs will attract each other. The displacement of arc 2 caused by the electromagnetic force of arc 1 can be expressed as [39]:

(5) $$l_{L}=\frac{I_{T}L_{L}^{2}}{{2I}_{L}D_{E}}$$

It can be seen from the above formula that when the arc length *L*_L_ and the arc distance *D*_E_ are constant, the displacement *l*_L_ is in a fixed ratio *n* to $I_{T}/I_{L}$, with *n* = $L_{L}^{2}/{2D}_{E}$. The smaller the current of the arc itself, the greater the adjacent arc current, and the greater the displacement.

**Figure 13:**
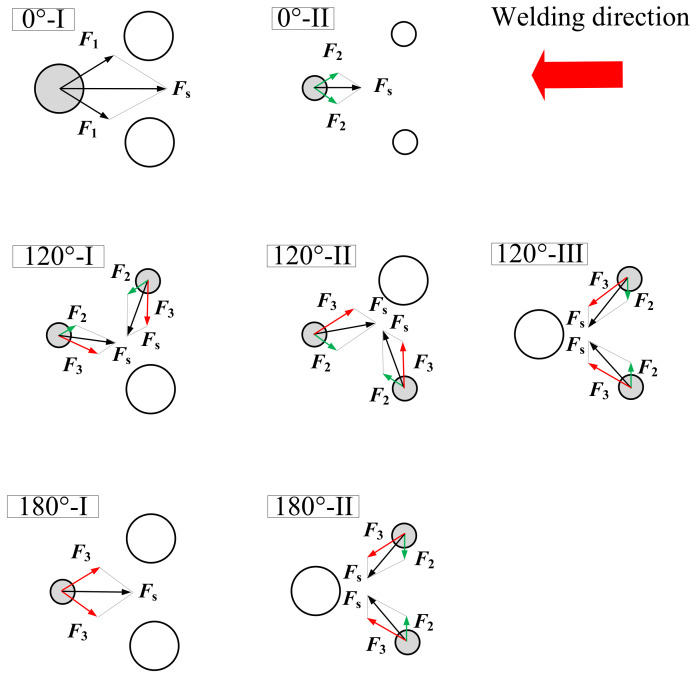
Typical arc states at different pulse phase angles for the triple-wire welding.

For the triple-wire pulse welding, the arc presents multiple states due to the variety of arc numbers and current phase. Figure 13 schematically shows various typical arc states for the phase angles of 0°, 120°, and 180° in a top down view. The large circle represents the arc in pulse peak phase, and the small circle represents the arc in a base phase. For example, the 120°-I state in the figure represents that the trailing wire arc 2 is at a pulse peak current of 540 A, and the guide wire arc and the trailing wire arc 1 are at a pulse base current of 40 A. The electromagnetic forces are also given in the figures for the arcs with small current, since these are more prone to break due to the electromagnetic force of adjacent arcs.

In Figure 13, *F*_1_, *F*_2_, and *F*_3_, respectively, represent the electromagnetic forces between the peak current arc and the peak current arc, between the base current arc and the base current arc, and between the base current arc and the peak current arc. The forces between arcs are vectors and obey the principle of vector synthesis. Taking the arc in the 120°-I state as an example, the leftmost arc is affected by the force *F*_2_ caused by the adjacent base current arc and the force *F*_3_ caused by the adjacent peak current arc. Known as *F*_ML_ = *m*$I_{L}I_{T}$, then *F*_2_ is 40·40·m, and *F*_3_ is 540·40·m. Since *F*_2_ and *F*_3_ are at an angle of 60°, their resultant force *F*s is equal to 14·40^2^·m. The arc offset is caused by external force, so the direction of arc offset is the same as the direction of the resultant force *F*s, and the calculation method for the arc offset *L*s is similar with that for the arc force *F*s.

Table 5 shows the pressure difference *P*_ML_, electromagnetic resultant force *F*_s_ and arc offset *L* for the arcs in different states. The greater the arc offset, the easier the arc is to break. Comparing the values for the 120° and 180° phase angles, one can see that the arc force and arc offset at the 180°-I state are greater than those in the other states, so the arc is easier to break in this case, which is consistent with our experimental results.

However, for the 0° phase angle, the situation becomes complicated. The theoretical analysis shows that the arc is the least likely to break at 0° since its arc offset is the smallest, while the experimental results show that the arc was more likely to break at 0° than at 120°. After carefully checking the electrical signals and high-speed camera photos, we found that the reason lies in the time delay between current pulses. In our experimental setup, the pulse trigger signal was sent by the main welding machine, and there was a slight time delay of about 0.3 ms from the main machine to the two slave welding machines (See Figure 4). Not only does the pulse rise first appear on the guide weld wire arc, but also the pulse current fall first appears on it. In the current fall stage, due to the time delay, the guide wire current was significantly smaller than the two trailing wire currents, e.g., *I*_1_ = 55 A, *I*_2_ = 182 A, and *I*_3_ = 146 A at *t* = 0.813 s, as shown in Figure 4. As a consequence, due to the electromagnetic action of the two strong trailing wire arcs, the guide wire arc was broken. This situation is very similar to the arc interruption at 180° (one weak arc and two strong arcs). Nevertheless, because the arc current in this case was still weaker than that at 180°, the probability of arc interruption at 0° was less than that at 180°, just as our experimental results have shown. In the future, we hope to solve the problem of arc interruption at the 0°phase angle by eliminating the time delay between current pulses.

## 5. Conclusions

This paper presents the experimental investigation of an integrated circular triple-wire pulse GMAW of Q960E high-strength steel for construction machinery. The arc stability has been emphatically studied for three typical pulse phase angles (0°, 120°, and 180°). In addition, in order to verify its usability in construction machinery and determine the ideal heat input range, different heat input (0.99, 1.26, 1.56, and 1.89 kJ/mm) were used to weld a 9 mm thick Q960E plate, which is typically used for the arm of construction machinery. The following conclusions are drawn:

1. For the three phase angles, the arc stability from bad to good was as follows: 180°, 0°, 120°. When the phase angle was 180°, its arc stability was the worst, because the guide wire arc was easy to break in the base current stage due to the strong electromagnetic action of the two pulse trailing wire arcs; when the phase angle was 0°, the guide wire arc also occasionally broke in the current fall transition stage because its current was weaker than that on the two trailing wires due to pulse delay, so the arc stability was still not ideal; when the phase angle was 120°, its arc stability and weld appearance was the best and ideal.
2. In the heat input range of 0.99–1.89 kJ/mm, the weld zone of the welded joint was dominated by acicular ferrite, which was mixed with a small amount of ferrite side plate. With the increase of heat input, the needle blade size of the acicular ferrite increased, and the content of the ferrite side plate in the weld increased. With the increase of heat input, the microstructure of the heat-affected zone transformed from lath martensite to a mixed structure of lath martensite and lath bainite and then to lath bainite. The tensile strength of the welded joint showed a decreasing trend, and the impact toughness of the weld zone decreased with the increase of heat input, while the impact toughness of the heat-affected zone increased first and then decreased.
3. When the preheating temperature was 100–120 °C, the welding heat input was controlled at 1.26–1.56 kJ/mm, the groove angle was 60°, the weld zone was dominated by acicular ferrite, and the coarse-grained zone of the heat-affected zone was a mixed structure of lath martensite and lath bainite. The tensile strength of the welded joint reached 85% of the base metal, and the impact toughness was above 62 J, which can meet the requirements of construction machinery for joint performance. This indicates the usability of the triple-wire welding in construction machinery.

In spite of the preliminary investigation of the integrated circular triple-wire GMAW process, the present work should build a basis for further studies, such as the improvement of arc stability at the 0° phase angle by adjusting the pulse-triggered mode, the controlling of the weld microstructure and performance by adding specific chemical components to one or several wires, and the application of this method to non-ferrous metals (e.g., aluminium alloy) or thicker plates. These improvements will be done in our forthcoming work.

## Figures and Tables

**Table 1:** Pulsed current waveform parameters for individual arc.

Electrical Parameters	Values
Peak current *I*_p_, A	540
Transition current *I*_t_, A	110
Base current *I*_b_, A	40
Peak time *t*_p_, ms	2.2
Transition time *t*_t_, ms	3.8

**Table 2:** Chemical compositions of the Q960E steel and THQ80-1 wire (wt%).

	C	Mn	Si	P	S	Cr	Ni	Mo	Ti	Cu	Nb	Ni	Fe
Q960E	0.16	1.24	0.31	0.014	0.003	0.39	0.61	0.47	0.021	0.02	0.019	0.61	Bal.
THQ80-1	≤0.10	1.50–1.80	0.40–0.80	≤0.02	≤0.02	0.20–0.40	1.30–1.60	0.25–0.50					Bal.

**Table 3:** Welding conditions for bead-on-plate welding.

Welding Parameters	Values
Preset current *I*_set_, A	140
Preset voltage *U*_set_, V	24.0
Welding speed *v*, m/min	0.8
Shielding gas	82%Ar-18%CO_2_
Gas flow, L/min	30
Phase differences	0°, 120°, 180°

**Table 4:** Welding parameters for the butt welding.

Test Number	Preset Current *I*_set_/A	Preset Voltage *U*_set_/V	Welding Speed *v/*mm/s	Heat Input *E*/(kJ/mm)	Weld Pass
1	120	22	8	0.99	3
2	140	24	8	1.26	2
3	160	26	8	1.56	2
4	180	28	8	1.89	2

**Table 5:** Arc interruption analysis for the triple-wire welding arcs in different current states.

Arc State	Current	Pressure Difference, *P*_ML_	Electromagnetic Resultant Force, *F*s	Arc Displacement, *L*_S_
0°-I	540-540-540 A	540^2^·*k*	315.7·40^2^·*m*	1.732·*n*
0°-II	40-40-40 A	40^2^·*k*	1.732·40^2^·*m*	1.732·*n*
120°-I	40-540-40 A	40^2^·*k*	14.0·40^2^·*m*	14.0·*n*
120°-II	40-40-540 A	40^2^·*k*	14.0·40^2^·*m*	14.0·*n*
120°-III	540-40-40 A	40^2^·*k*	14.0·40^2^·*m*	14.0·*n*
180°-I	40-540-540 A	40^2^·*k*	23.4·40^2^·*m*	23.4·*n*
180°-II	540-40-40 A	40^2^·*k*	14.0·40^2^·*m*	14.0·*n*

## Data Availability

The data presented in this study are available on request from the corresponding author.
